# The interplay between genomic copy number variants, sleep, and cognition in the general population.

**DOI:** 10.21203/rs.3.rs-5200475/v1

**Published:** 2025-03-19

**Authors:** Cecile Poulain, Rackeb Tesfaye, Elise Douard, Martineau Jean-Louis, Zohra Saci, Aurelie Labbe, David C Glahn, Laura Almasy, Mayada Elsabbagh, Guillaume Huguet, Sebastien Jacquemont

**Affiliations:** 1 Centre Hospitalier Universitaire Sainte-Justine Research Center, Montréal, Quebec, Canada; 2 Bioinformatics Graduate Program, Department of Biochemistry and Molecular Medicine, Université de Montréal, Montréal, Quebec, Canada; 3 Department of Neurology and Neurosurgery, McGill University, Montreal Neurological Institute, Azrieli Centre for Autism Research, Montréal, Quebec, Canada; 4 Department of Neuroscience, Université de Montréal, Montréal, Montréal, Quebec, Canada; 5 HEC Montreal, Department of Decision Sciences, Université de Montréal, Montréal, Quebec, Canada; 6 Department of Psychiatry, Boston Children’s Hospital, Boston, MA, USA; 7 Department of Psychiatry, Harvard Medical School, Boston, MA; 8 Department of Biomedical and Health Informatics, Children’s Hospital of Philadelphia, Philadelphia, PA, USA; 9 Department of Pediatrics, Université de Montréal, Montréal, Québec, Canada

## Abstract

Genomic Copy Number variants (CNVs) increase risk for neurodevelopmental disorders (NDDs) and affect cognition, but their impact on sleep remains understudied despite the well-established link between sleep disturbances, NDDs, and cognition.

We investigated the relationship between CNVs, sleep traits, cognitive ability, and executive function in 498,852 individuals from an unselected population in the UK Biobank.

We replicated the U-shape relationship between measures of cognitive ability and sleep duration. The effects of CNVs on sleep duration were evident at the genome-wide level; CNV-burden analyses showed that overall, CNVs with an increasing number of intolerant genes were associated with increased or decreased sleep duration in a U-shape pattern (p < 2e^−16^), but did not increase risk of insomnia. Sleep duration only marginally mediated the robust association between CNVs and poorer cognitive performance, suggesting that sleep and cognitive phenotypes may result from pleiotropic effects of CNVs with minimal causal relationship.

## INTRODUCTION

Genomic Copy Number Variants (CNVs) are deletions and duplications over 1000 base pairs. CNVs are well-established biological factors strongly linked to susceptibility to Neurodevelopmental disorders (NDD) and are associated with negative effects on cognition, even in individuals from unselected populations who do not meet criteria for an NDD diagnosis^1^. Cognitive ability is one of the most commonly assessed quantitative traits in pediatric clinics, as it helps predict outcomes and adaptive skills in children with neurodevelopmental symptoms^2^. Many studies have shown that the effect size of CNVs on cognitive ability is related to their effect size on liability for NDDs^1^. As such, measuring effect size on cognitive ability provides a nuanced insight into the severity of genetic variants associated with NDD risk in general population cohorts where the prevalence of NDD is low.

Measures of intolerance to haploinsufficiency (i.e., genetic variants that inactivate protein-coding genes) of genes encompassed in CNVs have emerged as the best metrics to predict the effect size of multigenic CNVs (genome-wide) on cognition^3^, risk for NDDs and psychiatric conditions such as autism, schizophrenia, Major Depressive Disorder (MDD), bipolar, and Obsessive-Compulsive Disorder (OCD) ^4–6^.

Despite the fact that sleep disturbances are some of the most common comorbidities across NDDs (i.e., up to 86% of individuals with NDDs are reported to experience sleep problems^7^) they remain understudied in individuals who carry genetic risk for NDDs.

Indeed, sleep disturbances have long been linked to cognitive difficulties across the developmental lifespan in individuals with and without a diagnosis of NDD or psychiatric condition^8–13^. It has been posited that disturbed sleep may contribute to exacerbating cognitive impairments in NDD groups, in particular executive functioning^14^, which enables goal-directed thought and behavior. In recent years, support for an inverted U-shape association between sleep duration and cognition has emerged^15–18^. Such that individuals who have shorter or longer sleep compared to the average duration in their cohort, experience poorer cognitive performance. Studies documenting that sleep problems are associated with genetic NDDs^19^ are limited to specific well-known recurrent CNVs, like 16p11.2, 22q11.2, 15q11.2–13.1^20–22^. Mouse models have also demonstrated evidence for such relationships, including sleep alterations in 16p11.2 deletions mouse models and circadian rhythm disruptions in 17p11.2 deletion mouse models^23^. However, the relationship between rare large effect genetic risk for NDDs, cognitive deficits, and sleep traits is still understudied.

### Knowledge Gap:

The effects of NDD-associated CNVs on sleep traits have not been investigated genome-wide. Understanding whether sleep disturbances may mediate some of the well-known effects of CNVs on cognitive abilities would provide insights into the mechanisms underlying genetic risk for neurodevelopmental disorders.

**Our overarching aim** is to investigate the relationship between sleep and cognition and the effects of CNVs on both traits in an unselected population.

Specifically, we aim to : (i) Examine the relationship between self-reported and actigraphy-derived measures of sleep with cognition and executive functions in the UK Biobank (UKBB); (ii) Investigate if recurrent CNVs associated with cognition and NDD risk are associated with sleep quantity and quality as well as lower cognition in the UKBB; (iii) Investigate if sleep traits mediate the relationship between CNVs and cognitive ability.

To reach these goals, we investigated all CNVs genome-wide in 498,852 individuals from an unselected population (UKBB) with measures of general cognitive ability, executive functioning, self-reported, and accelerometer-derived measures of sleep. Measures of general cognitive ability and executive functioning (EF) were used to evaluate whether associations remain robust across cognitive domains. As such, this large sample provided an opportunity to disentangle the relationship between genes, sleep and cognition.

## RESULTS

### Associations between sleep and cognition in the UKBB.

Participant characteristics can be found in Table 1. Measures of sleep duration examined using GAM smooth line exhibited an inverted U-shaped relationship with cognition (eFigure1), hence we fitted a quadratic model. The latter demonstrated a significant relationship between sleep duration, general cognitive ability, as well as executive functioning (Figure 2). The only non-significant association was between accelerometer-derived sleep duration and the Tower Rearranging task, which had the smallest sample size. (eTable3). Quadratic trends demonstrated that any deviation from the mean sleep duration (7 to 7.5h) was associated with worse cognitive performance. A Likelihood Ratio Test, showed that the quadratic model outperformed linear models (eTable3). Moreover, this quadratic trend remained when restricting the analysis to self-reported sleep duration and cognitive variables administered at the same time-point and when removing individuals above 65 years of age as well as those taking any psychiatric or sleep medication (eTable1, eTable3). In contrast with sleep duration, sleep efficiency (derived from accelerometer data) was not associated with general cognitive ability or executive functioning measured by the tower rearranging task, and a weak association was only observed with trail making task (β= 0.20, p= 4.60^−04^, eTable4). These observations remained unchanged when removing individuals with medication and above 65 years (eTable4).

The reporting of insomnia traits was also associated with lower measures of general cognitive ability (β= −0.05, p=7.70^−31^, eTable4). This effect size was unchanged when the analysis was restricted to probable insomnia and cognitive assessments collected at the same time point (β= −0.03, p=2.27^−04^, eTable4) and when removing individuals taking medication as well as the elderly (β= −0.04, p= 1.24^−11^, eTable4). Executive functioning measured by the trail making task and tower rearranging task (eTable4) showed weak to no association with self-reported insomnia.

### Associations between recurrent CNVs, sleep and cognition

17,162 individuals in the UKBB carried a CNV at 29 genomic loci previously associated with varying effects on cognitive ability and risk for NDD were identified in the UKBB (eTable2). As expected, a large proportion of CNVs (thirteen) negatively affected general cognitive ability with mild to large effect sizes, except for the positive effects observed for the deletion of *ZNF92* (β= 0.06, p=1.88^−02^, Figure 3, eTable5) previously reported to have a protective effect on schizophrenia^35^. The 17p12 deletion (HNPP syndrome) was also found to increase performance on the tower rearranging task (β= 0.74, p=1.55^−02^, Figure 3, eTable5). Four CNVs, three deletions (10q11.21q11.23, 15q11.2, 22q11.2 distal) and one duplication (16p11.2 distal), showed significant moderate to large effects on sleep duration either self-reported or derived from the accelerometer (Figure 3, eTable5, Note: 22q11.2 distal deletions increased in self-reported and accelerometer-derived sleep duration). Interestingly, CNVs either decreased or increased self-reported and accelerometer-derived sleep duration. No CNVs were associated with sleep efficiency or probable insomnia (Figure 3).

### Genome wide effects of deletions and duplications on sleep and cognition in the UKBB

The analysis above was limited to recurrent CNVs observed in more than 10 individuals. It is likely, however, that these effects may be observed for a much broader spectrum of CNVs burden (genome-wide). We therefore used a previously published method to measure the average effects of CNVs weighted by intolerance to haploinsufficiency (measured by LOEUF). This method has been used to associate CNV burden with many phenotypes, including cognitive ability and risk for neurodevelopmental disorders. As previously reported^3,4,31^, there was a strong association between the weighted burden of CNVs and general cognitive ability (β= −0.04, p=5.61^−42^ for deletions; β= −0.0919, p=2.69^−33^ for duplications, eTable6).

Using GAM, we observed a U-shaped trend between sleep duration (self-reported) and the severity of CNVs measured by the $\Sigma \frac{1}{LOEUF}$. We therefore tested this association using a linear and quadratic model. The quadratic model showed a significant association for deletions and duplications (Figure 4a, 4b, eTable7). In contrast, the linear model showed no association and was outperformed (ANOVA : p=3.38^−22^ for deletions ; p=5.22^−25^ for duplications) by the quadratic model for deletions and duplications (Figure 4a, 4b, eTable7). Sensitivity analysis showed a strong association between sleep duration and the $\Sigma \frac{1}{LOEUF}$ (eTable7). Overall, this suggests that individuals carrying CNVs of increasing severity exhibit increasingly shorter or longer sleep duration. We did not identify any effects of CNVs burden (measured by LOEUF) on probable insomnia or accelerometer-derived sleep measures (eTable7).

We therefore stratified the coding genome into 4 categories with increasing intolerance to haploinsufficiency as measured by LOEUF (highly intolerant < 0.2, intolerant= [0.2;0.35[, mildly-intolerant = [0.35;1[, tolerant ≥ 1). Intolerant genes were associated with increasing negative effects on cognitive performance and increased deviation from the average self-reported sleep duration (7.1 hrs) in the UKBB (eFigure 3). Genes within the intolerant category were associated with worst performance on both executive functioning tasks, but overall results were less robust likely due to smaller sample sizes (eFigure3). These results remained unchanged after removing individuals taking medication and above 65 years (eFigure3).

To better understand the relationships between CNVs (based on $\Sigma \frac{1}{LOEUF}$), general cognitive ability and sleep duration (self-reported), we performed a mediation analysis using a linear model and the absolute measure of sleep duration (Figure 4c, 4d). The analyses suggested that the genome-wide effects of duplications and deletions on cognitive ability were only very mildly mediated (5% for each) by sleep duration (eTable8). In other words, there is a genetic impact on general cognitive ability and sleep duration, but the effects on these 2 traits are mostly independent. Results remained when removing individuals taking medication and > 65 years (eFigure4)

## DISCUSSION

This study represents the largest investigation of rare CNVs, sleep traits and cognition. We confirm the inverted U-shaped relationship between sleep duration and cognitive abilities as previously documented^15–18^. Rare CNVs previously associated with NDDs as well as the genome-wide CNV-burden (weighted by LOEUF) were associated with worse cognitive performance and sleep duration. Interestingly, the latter association was in a U-shape fashion and, therefore, only visible using a quadratic model or the absolute values of sleep duration. In contrast, CNVs showed no association with sleep efficiency and insomnia. Although the genome-wide burden of CNVs was associated with both sleep duration and general cognitive performance, the association with these two traits were largely independent, as shown by the mediation analysis.

### The U shape relationship between sleep duration, biological factors, and other phenotypes.

We replicated the inverted U-shape relationship between sleep duration and cognitive measures. Our results echo the findings in other studies that have reported a U-shape relationship between sleep duration and health^38,39^, psychiatric symptoms^40,41^, and mortality^42^. Sleep duration is often investigated as a linear effect, which may overlook U-shape relationships and may explain some of the conflicting results such as those reported between ASD and sleep duration^14^. Quadratic U-shape relationships have also been established between sleep duration and intermediate brain phenotypes. Brain volume of 46 cortical and subcortical regions were associated with deviation from the average sleep duration in a U-shape fashion^17^. The same study also showed that markers of cerebrovascular burden (i.e., white matter hyperintensities) show the same quadratic association with sleep duration. Interestingly, we also identified a U-shape relationship between genome-wide CNV burden and sleep duration, which could not have been identified without adding a quadratic term. The mechanisms underlying the U-shaped association in which both short and long sleep duration are associated with worse cognitive performance remain to be elucidated. One key question raised by previous studies is whether short and long sleep duration might be risk factors, early markers, or a result of cognitive deficits^16^. Our mediation analysis suggests that the association between CNVs and cognitive performance is not mediated by sleep duration. We therefore posit that deviation from average sleep duration may be an additional phenotype (rather than a causal factor) of biological factors (including CNVs) contributing to cognitive deficits and risk for psychiatric conditions.

### CNVs increasing risk for NDDs and decreasing cognition only mildly affect sleep traits.

Overall, our findings on the effects of individual recurrent CNVs on cognition are in line with previous research^43^. Our results further show that these effects are more robust for general cognitive ability, rather than specific tasks like executive functioning. We observed a positive effect on general cognition for the deletion of *ZNF92* which has been previously reported to have a protective effect on schizophrenia^35^. We also report for the first time a CNV associated with an improvement in executive functioning performance (17p12 deletions). This is consistent with the recent study reporting a protective effect of 17p12 deletions for psychiatric disorders [HR 0.4–0.8;^44^].

While genome-wide CNV burden showed a clear association with sleep duration, only a few specific recurrent CNVs tested in this study were associated with measures of sleep duration (self-reported sleep and accelerometer-derived measures), which may be in part related to the limited sample size for each recurrent CNV. The 22q11.2 distal deletions were found to increase sleep duration in both measures by over 20 minutes. This increase in sleep duration in 22q11.2 distal has not been previously documented to our knowledge. 16p11.2 distal duplications were shown to decrease accelerometer derived sleep duration by 40 minutes, but were not associated with self-reported sleep. This is in line with recent findings showing no differences in self-reported sleep duration between 16p11.2 carriers, family members and community controls^21^.

Overall, the associations between sleep duration and CNV-burden is consistent with the polygenic nature of this trait^45^ and suggest that, when deleted or duplicated, many genomic loci are related to variations in sleep duration. Our burden analysis (weighted by LOEUF) as well as our sensitivity analysis, stratifying genes based on their constraint score, shows that intolerant genes are more likely to alter sleep duration and cognitive abilities but this association is much stronger for cognitive ability. This may indicate that as opposed to cognitive ability, sleep may be linked to more restricted set of biological functions such as those identified through GWAS of common variants^45,46^. Indeed, LOEUF values reflect genetic fitness, which may only be weakly related to mechanisms involved in sleep traits.

### CNVs affecting intolerant genes are not associated with insomnia.

Interestingly, despite evidence of a considerable genetic contribution (heritability 38–59% ^47^ ) to insomnia, and the previously reported genetic correlation between insomnia and cognitive ability^45,46^, we did not detect an association between insomnia and specific recurrent CNVs or with the genome-wide burden of CNVs (weighted by LOEUF). Recent large GWAS^45^ of insomnia have reported many associated loci, and a strong genetic correlation with depression and anxiety. The latter may in part explain the lack of association between insomnia and CNVs, which show only weak association with anxiety and depression^48,49^. This lack of association may also be due to the differing impacts of rare versus common variants on these traits or a recruitment bias in the UKBB. In contrast, CNVs showed a clear association with sleep duration. This is consistent with the genetic correlation between sleep duration, schizophrenia, and educational attainment, which is not seen between depression and anxiety. This is also in line with the fact that CNVs are also strongly associated with schizophrenia and educational attainment^35,43^.

#### Limitations

Given the nature of the UKBB protocol, not all sleep and cognitive variables were collected at concurrent time-points. Of note, our results were unchanged when restricting analyses to sleep and cognitive measures collected at the same time-point. Previous findings also demonstrate that cognitive performance remains stable during follow-up in the UKBB^50^. Although sleep duration was mostly self-reported, when available, an accelerometer confirmed these reports. Similar to previous studies in UK biobank and other cohorts, we do not attempt to infer any causal relationships between sleep, cognitive or other phenotypic measures.

## CONCLUSION

Our study showed that the genome-wide burden of CNVs across a very large proportion is associated with decreased cognitive abilities and sleep duration in a U-shape fashion. While several previous studies have suggested that changing sleep duration may improve cognitive outcomes, our mediation analysis may suggest that both sleep duration and cognitive ability are two phenotypes with little causal relationship and may result from pleiotropic effects of genetic and non genetic factors. These results add to the emerging complexity of the quadratic relationship between sleep duration and multiple health and behavior outcomes.

## METHODS

### Participants:

Data was analyzed from the UK Biobank study, which has been extensively described elsewhere^24^. Over 500,000 individuals from the United Kingdom aged 37–74 were recruited between 2006–2010 (initial assessment visit). Information was also obtained from additional assessment visits between 2012–2020, along with online follow-up questionnaires.

### Cognitive measures:

#### General Cognitive ability.

We computed a Gfactor score using Principal Component Analysis when possible. To further maximize the inclusion of participants with completed cognitive tasks, Gfactor scores were pooled with fluid intelligence scores completed during the first available time-point. For further details, see *Supplementary eMethods*.

#### Executive functioning.

The cognitive shifting component of executive functions was measured using the Trail Making Task administered at initial assessment and online. Planning abilities were measured using accuracy on the Tower Rearranging Task available only for the initial assessment. See *Supplementary eMethods*.

All cognitive scores described were transformed into z scores adjusting for age and sex.

### Sleep measures:

#### Self-report questionnaires.

We analyzed two sleep traits self-reported during initial assessment:

- Sleep duration (1106); the number of hours spent sleeping over a 24hr period (including naps) responses were made in 1-hr increments. Extreme responses of less than 3hrs and more than 12hrs were excluded as recommended by previous studies^25,26^
- Probable insomnia (1200); participants responding “sometimes” or “usually” to “Do you have trouble falling asleep at night, or do you wake up in the middle of the night?”, were coded as having probable insomnia. Participants responding “never/rarely” were coded as controls, while participants indicating “prefer not to answer” were removed.

#### Accelerometer.

A subset of participants were invited to wear a triaxial accelerometer device (Axivity AX3) between 3 to 10 years after the initial study assessment. Individuals were excluded based on UKBB quality checks previously described^26,27^, we further excluded individuals wearing the device for <5days and those with extreme short (<3hrs) or long (>12hrs) mean sleep duration^25,26^. Detailed descriptions of raw accelerometer data is available elsewhere^27^. Converted and processed raw data using the R package GGIR was retrieved from UKBB return 1862^33^.

Two Accelerometer derived sleep traits were analyzed:

- Sleep duration; calculated as sleep episodes within the Sleep Period Time (SPT) lasting more than 5 minutes with no detected change > °5 associated with the z-axis of the accelerometer. Duration for all sleep episodes were summed and averaged across nights available.
- Sleep efficiency; calculated as a ratio of sleep duration divided by the time elapsed between the beginning of the first inactivity bout and end of the last inactivity bout (SPT window).

The ratio represents the mean of all nights available.

#### *Medications* (20003).

Participants self-reporting medication use that interfere or likely interfere with sleep and circadian rhythms^25^ were excluded in sensitivity analysis (eTable1).

### Genetic information

Genetic information was retrieved from the UKBB. Genotyping collection, quality control and imputation procedures have been described in detail^28^. Genome wide-analyses controlled for ancestry. Ancestry was computed by the UKBB using a PCA yielding 10 distinct ancestral groups (22009).

CNVs were called using PennCNV^29^ and QuantiSNP^30^ and filtered based on published methods^3,31^. Pipeline procedures can be accessed on GitHub: https://martineaujeanlouis.github.io/MIND-GENESPARALLELCNV/#/ and final CNV calls can be requested from UKBB [Return ID: 3104]. For further information, see *Supplementary eMethods.* CNVs were annotated using Gencode V19 annotation (the reference release for hg19 Human genome release) with ENSEMBL gene (https://grch37.ensembl.org/index.html). Each gene was annotated when it’s possible with*“*Loss-of-function observed/expected upper bound fraction” (LOEUF)^32^. It is a continuous score ranging from 0 to 2 and used to measure haploinsufficiency. Values below 0.35 are suggestive to be highly intolerant to haploinsufficiency.

Finally, we identified recurrent CNVs related to NDDs and negative effects on cognition from previously published studies^33–37^. CNVs were only analyzed if more than 10 carriers in the UKBB were identified. The CNVs identified are described in eTable2.

### Data analysis

**Figure 1.**
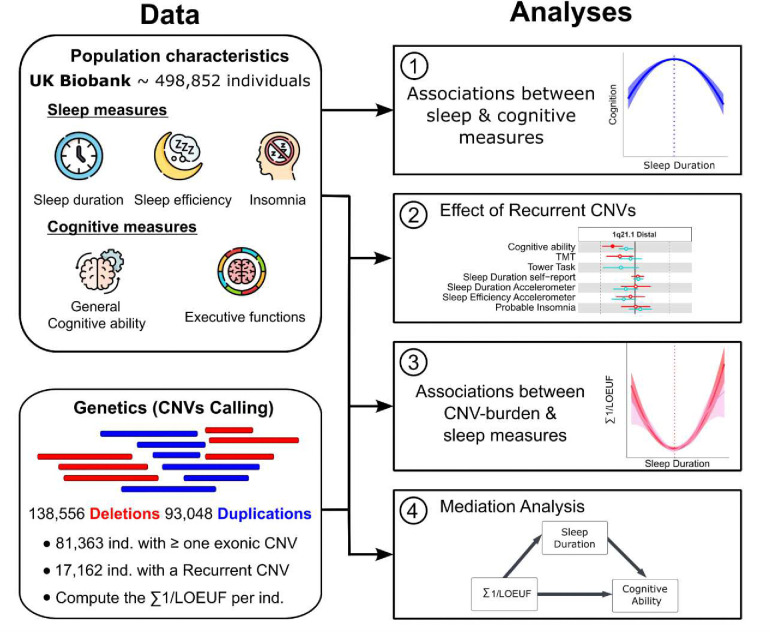
Methodology

**Figure 2.**
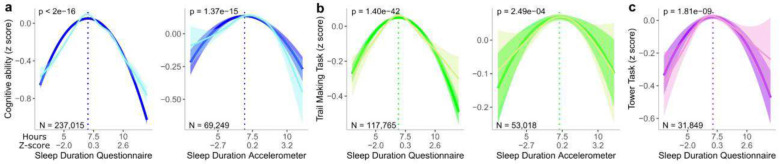
U-shape/quadratic associations between sleep duration and cognition performance. Fitted curves of the Quadratic model associating cognition and sleep duration (dark line). The light line represents the GAM model. The dotted vertical line indicates the vertex of the parabola. a) Relationship between general cognition aggregated across visits and sleep duration (self-reported on the left and accelerometer on the right). b) Relationship between executive functioning measured by the trail-making task administered in person and online and self-reported sleep duration (self-reported on the left and accelerometer on the right). c) Relationship between executive functioning measured by the tower rearranging task administered in person and self-reported sleep duration (Note: this relationship was not significant with accelerometer-derived sleep duration). Blue=General cognitive ability, Green=Trail making task, Purple=Tower task. X-axis: durations are in hours (first line) and z-scored duration (second line). Y-axis: z-scored cognitive ability.

**Figure 3.**
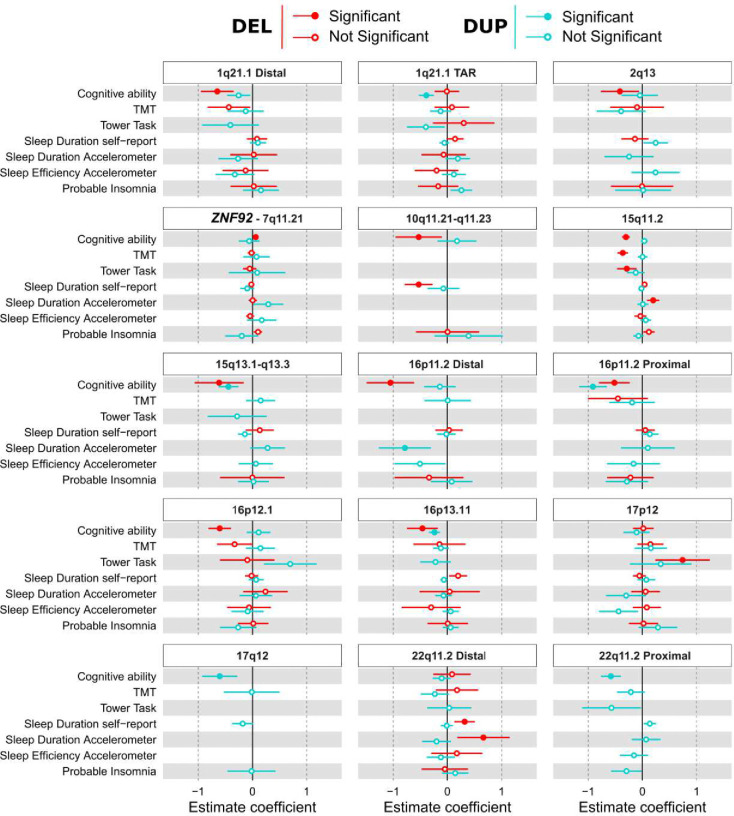
Associations between CNVs, sleep, and cognitive measures. Forest plots depicting the associations between specific recurrent CNVs observed in >10 carriers (red=deletion;blue=duplications) and 7 cognitive and sleep measures. Only CNVs with a significant association (FDR < 0.05) with at least one trait are shown. The Y-axis represents the cognitive and sleep traits. The X-axis represents the estimate (z-score) of the linear models associating CNVs and traits. Abbreviation, TMT: Trail Making task.

**Figure 4.**
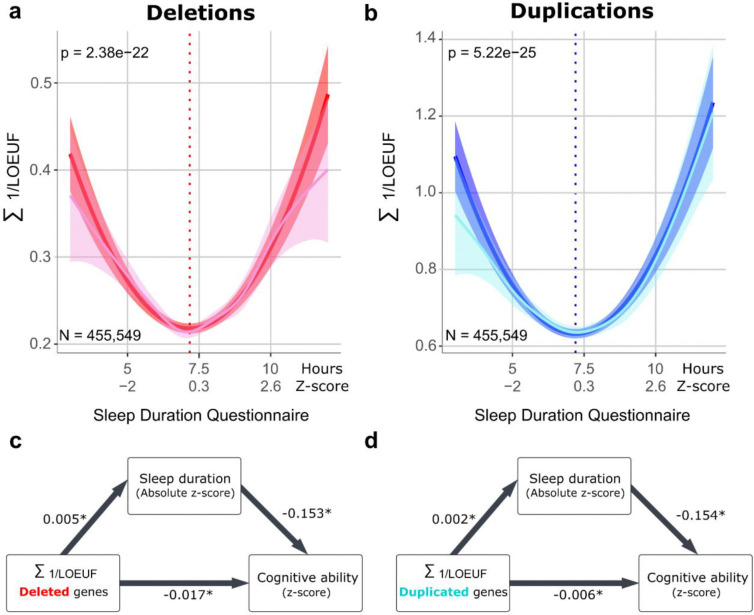
Associations between haploinsufficiency, sleep and cognition ***a,b*** *represent the fitted curves of the quadratic model associating CNVs measured by their sum of 1/LOEUF (for* ***(a)*** *deletions and* ***(b****) duplication) with self-reported sleep duration (dark line). The light line represents the GAM model. The dotted vertical line indicates the vertex of the parabola.* ***c-d*** illustrates the results of the mediation analysis between the sum of LOEUF (for deleted and duplicated genes), sleep duration, and cognitive ability ( * p-value < 2e^−16^*). The Average Causal Mediation Effect effect was 0.005* ✕ *−0.153 = −0.0008 (p < 2e-16) for deletions and 0.002* ✕ *−0.154 = −0.0003 (p < 2e-16) for duplications. The proportion mediated was 4.5% for deletions and duplications.*

An overview of the different analyses is illustrated in Figure 1.

All analyses were performed with R 4.3.1

#### Associations between sleep and cognition.

The relationship between cognitive variables (general cognitive ability, trail making and tower rearranging task) and sleep duration (self-report and accelerometer derived) was first plotted with a General Additive Model (GAM) smooth line using the R ‘gam’ package. To confirm plotted relationships and to test a priori inverted U-shape relationships published, linear and quadratic models were run for each cognitive variable, with sleep duration as the independent variable. Likelihood ratio test (ANOVA for nested models) was applied to compare significant differences between models, using higher adjusted R-squared values and sleep duration p-values to select the optimal model. Sleep duration associated with the highest cognitive performance (x-coordinate of a parabola’s vertex) was calculated as: $-\frac{B}{2A}$. A refers to the estimated coefficient for the squared sleep duration variable and B the duration coefficient for the linear term. Separate general linear models were used to test the relationship between sleep efficiency and probable insomnia, with each cognitive variable. For all models, age at which sleep traits were reported was controlled for, along with the 10 principal components. Subsequent sensitivity analyses included: restricting comparisons between sleep and cognitive variables reported at the same initial assessment and removing individuals taking medication and above 65 years.

#### Effects of individual CNVs on sleep and cognition.

Separate linear regression models were applied to investigate associations between independent CNVs of interest with each cognitive and sleep dependent variable, with the exception of insomnia, for which a logistic regression was used. In all models, carrier status for each individual CNV was the independent variable, with carriers as the reference group. All models controlled for age, sex and the 10 first principal components. In order to correct for multiple testing, FDR correction was applied within each phenotype, separately for deletions and duplications.

#### Effects of haploinsufficiency on sleep and cognition.

##### Based on LOEUF categories:

The effect size of genes deleted and duplicated were stratified by score of intolerance to Loss of Function variants. For each individual, the number of genes deleted or duplicated was assessed in four categories defined across the range of LOEUF values: highly intolerant genes (0.03 ≤ LOEUF < 0.2), intolerant genes (0.2 ≤ LOEUF < 0.35), moderately intolerant genes (0.35 ≤ LOEUF < 1), and tolerant genes (1 ≤ LOEUF ≤ 2).

Regressions were performed as follows, separately for deletions and duplications:

$$Y_{i}={\text{phenotypic}\;\text{measure}}_{i}∼\beta_{0}\;\;+\beta_{1}\cdot \Sigma {\left({\text{genes}}_{i,\text{inside}\;\text{category}}\right)}_{\text{DEL/}\;\text{DUP}\;}\;\;+\beta_{2}\cdot \Sigma {\left({\text{genes}}_{i,\text{outside}\;\text{category}}\right)}_{\text{DEL/}\;\text{DUP}\;}\;\;+\beta_{3}\cdot \text{age}\;\;+\beta_{4}\cdot \text{sex}\;\;+\text{first}\;10\;\text{principal}\;\text{components}$$

Where $\Sigma ({\text{genes}}_{i}$ inside the ${\text{window}}_{j}{)}_{\text{DEL/DUP}}$ is the number of genes deleted (DEL) or duplicated (DUP) for the individual i inside the LOEUF category j. $\beta_{0}$, $\beta_{1}$, $\beta_{2}$, $\beta_{3}$ and $\beta_{4}$ are the vectors of coefficients for fixed effects. Linear regressions were applied for all sleep and cognitive measures, with the exception of probable insomnia (logistic regression).

##### Based on aggregate LOEUF Values :

We computed the $\Sigma \frac{1}{LOEUF}$ separately for all genes included in deletions or duplications to investigate the relationship between genetic factors and sleep duration measures. We applied the same steps, as previously for investigating U-shape association between sleep and cognition : (i) fitting a General Additive Model (GAM) smooth line, (ii) run linear and quadratic models (iii) Compare linear and quadratic models using likelihood ratio test.

Separate linear models were used to test the relationship between genetic factors and other sleep/cognitive variables. All models were controlled for age, sex and the 10 principal components.

#### Mediation analyses.

To investigate if sleep traits mediate the relationship between increased haploinsufficiency measured by LOEUF (independent variable) and cognitive ability (dependent variable), indirect effects were tested using the R ‘mediation’ package. Bootstrap procedures were applied, with indirect effects computed from 1000 imputations. Models were computed separately for $\Sigma \frac{1}{LOEUF}$ in deletions and duplications. The “Prop.mediated” output shows how the mediator (sleep trait) influences the relationship between LOEUF and cognitive factors.

## Figures and Tables

**Table 1.** Sleep and cognitive characteristics in the UKBiobank

Variable	N individuals	Mean age (SD)	N females (%)	Mean (SD)
Probable Insomnia (self-report)	358,078 without insomnia	56.67 (8.22)	184,722 (52)	---
	139,300 with insomnia	58.01 (7.67)	85,703 (61)	---
Sleep Duration (Self-report)	497,378	57.07 (8.09)	270,425 (54)	7.15 hours (1.09)
Sleep Duration (accelerometer)	92,587	62.42 (7.83)	52,217 (56)	7.28 hours (0.86)
Sleep Efficiency (accelerometer)	92,587	62.42 (7.83)	52,217 (56)	0.76 (0.07)
General cognitive ability	238,351	59.03 (8.58)	129,836 (54)	0 (1)
Trail Making Task	121,117	60.48 (8.28)	65,947 (54)	0 (1)
Tower Rearranging Task	32,707	55.30 (7.53)	16,813 (51)	0 (1)
